# Drs. T. J. Crow

**Published:** 1871-09

**Authors:** H. J. Royal, W. H. Burr, M. A. Lowrance, O. J. Bond

**Affiliations:** Committee.; Committee.; Committee.; Rec. Sec'ty Southern Dental Association.


### OBITUARY NOTICES.

We regret to announce the deaths of two prominent members of the dental profession in the South, Drs. T. J. Crow, of Macon, Georgia, and Warren Johnson of Savannah, Georgia.

During the meeting of the Southern Dental Association, held in Charleston, S. C., in April 1871, the Committee on Memorials offered the following resolutions, which were unanimously adopted:

The Committee appointed to draft suitable resolutions in reference to the death of Dr. T. J. Crow, of Macon Ga., and Dr. Warren Johnston of Savannah Ga., beg leave to offer the following:

*Whereas*, it has pleased Almighty God to take from us two of our beloved brethren, and whereas, it not only becomes us, but it is our mournful duty, to pay a worthy tribute to the memory of those who have been so long identified with us, therefore,

*Resolved*, 1*st*, That in the death of our brethren we recognize the hand of an All-wise Providence, and though with grief we mourn their loss we bow with submission to the decree that has deprived us of those who won our highest esteem by their devotion to the interests of our profession, and who endeared themselves to us by their kindness of heart, which rendered them ever ready to alleviate the sufferings of humanity.

*Resolved*, 2*nd*, That we give expression to our fervent trust, that *our loss has been their gain*, inasmuch as they were ever known as faithful dentists, thorough gentlemen, and upright citizens.

*Resolved*, 3*rd*, That a page in the book of minutes of the Association be dedicated to their memory.

*Resolved*, 4*th*, That a copy of these resolutions be forwarded to the several Dental Journals for publication, and, also, to the relatives of our deceased brethren, with the assurance of our heartfelt sympathies in their bereavement.

H. J. Royal.

W. H. Burr. } *Committee.*

M. A. Lowrance.

O. J. Bond, Rec. Sec'ty Southern Dental Association.

